# Implementing an outpatient clinical trial on COVID-19 treatment in an emergency epidemic context: a mixed methods study among operational and research stakeholders within the Coverage trial, Bordeaux (France)

**DOI:** 10.1186/s13690-022-00999-9

**Published:** 2022-12-03

**Authors:** Carine Grenier, Macha Loniewski, Mélanie Plazy, Racha Onaisi, Marie-Hélène Doucet, Jean-Philippe Joseph, Alexandre Duvignaud, Denis Malvy, Xavier Anglaret, Joanna Orne-Gliemann, the Coverage study group

**Affiliations:** 1 University of Bordeaux, Bordeaux, France; 2 National Institute for Health and Medical Research (INSERM) UMR 1219, Bordeaux Population Health Research Centre, Bordeaux, France; 3 Research Institute for Sustainable Development (IRD) EMR 271, GHiGS, Bordeaux, France; 4 Department of General Practice, University of Bordeaux, Bordeaux, France; 5 Department of Infectious Diseases and Tropical Medicine, Division of Tropical Medicine and Clinical International Health, CHU Bordeaux, Bordeaux, France

**Keywords:** Covid, Treatment, Ambulatory, Perceptions, Implementation

## Abstract

**Background:**

The emergency set-up and implementation of outpatient clinical trials on epidemic emerging infectious diseases such as COVID-19 raise many issues in terms of research structuration, regulations, and health systems organization. We aimed to describe the experience and points of view of different stakeholders involved in a French home-based outpatient trial on COVID-19 and to identify the early barriers and facilitators to the trial implementation.

**Methods:**

We conducted a mixed-methods study in July 2020. A self-administered questionnaire was emailed to 213 clinical, operational and research stakeholders involved in the Coverage trial; individual semi-directed interviews were conducted among 14 stakeholders. Questionnaire data and written interview notes are presented together by key theme.

**Results:**

One hundred fifty six stakeholders responded to the questionnaire. 53.4% did not have prior experience in clinical research. The motivation of most stakeholders to participate in the Coverage trial was to feel useful during the pandemic. 87.9% agreed that the trial had an unusual set-up timeframe, and many regretted a certain lack of regulatory flexibility. Mobile medical teams and specific professional skills were perceived as instrumental for outpatient research.

**Conclusions:**

The implementation of a home-based outpatient clinical trial on COVID-19 was perceived as relevant and innovative although requiring important adaptations of usual professional responsibilities and standard research procedures. Lessons learned from the Coverage trial underline the need for improved networks between hospital and community medicine, and call for a dedicated and reactive outpatient research platform on emerging or threatening infectious diseases.

**Supplementary Information:**

The online version contains supplementary material available at 10.1186/s13690-022-00999-9.

## Introduction

Since the beginning of 2020, an unprecedented number of clinical trials were designed to identify efficient specific treatments for COVID-19 [1]. During the early stages of the pandemic, most COVID-19 clinical trials were conducted in inpatient settings, and among severe patients [2]. Most patients with COVID-19, including those at higher risk of severe disease, are diagnosed and followed up in primary-care and outpatient settings with mild or moderate symptoms; they need treatments that prevent clinical deterioration and can be managed in outpatient/ambulatory care. However, it is only more recently that outpatient trials were set-up to test the efficacy of investigational drugs administered at an early stage of the disease [3]. Many reasons may explain the paucity of clinical trials conducted in outpatient primary care settings, whether on COVID-19 or other diseases. Among them are: the fact that research interests of non-hospital-based physicians do not always align with priorities of funding agencies, the country specificities and structural factors (the United Kingdom, Australia or Canada for example are more advanced in primary care outpatient research than France, and the USA also have an important volume of publications in primary care [4, 5]), the limited number of general practitioners (GPs) trained on clinical research and Good Clinical Practices, or the limited number of senior researchers trained in outpatient clinical research [6]. Of note, young GPs seem to show increased interest in clinical research despite time and administrative constraints [7, 8]. Overall, in a context where there are constrained and competitive resources available for care and research in tertiary hospitals, where primary care is the entry point for most patients in the healthcare system, and where epidemics of highly communicable diseases/infections need to be contained rapidly, the need for outpatients and home-based clinical trials is increasing.

The implementation of clinical research in emergency and epidemic contexts, such as that of the COVID-19 pandemic, raises ethical, regulatory, administrative, logistical, social, cultural, political, and economic issues [9]. Despite growing research on the implementation of health programmes and policies, aimed at improving the adequacy of interventions and actions with the local needs and contexts, and at addressing barriers along the way, literature on the barriers and facilitators to implementing clinical trials remains scarce [10, 11]. When it comes to outpatient settings, it is scarcer or rather inexistent. There are reports from the field which highlight challenges with recruitment strategies and feasibility of hospital-based clinical and intervention trials [12, 13], which often lead to trial stop [14, 15]; guidelines for pilot feasibility trials also exist [16]. However, we did not find any published literature on the implementation of an outpatient clinical trial in an epidemic setting. Furthermore, although there is abundant literature on staff motivations and experiences of COVID-19 clinical services and patient care [17–19], we were unable to find any study exploring the experience and organisation of work among the different actors of COVID-19 research, or more largely of research on epidemic infectious diseases.

What is the acceptability of a COVID-19 outpatient trial from the perspective of key stakeholders in research and care? To what extent is such a trial adapted to their perceived needs and values? How do key stakeholders adjust and adapt their roles to an emergency epidemic context? What is the perceived feasibility of outpatient clinical research on COVID-19? What are the barriers or enabling factors to such research and what are the necessary adaptations in a context of an evolving epidemic and evolving care recommendations?

To address these key research questions, our study aimed to explore and describe the experiences, points of view and feelings of key stakeholders involved in a French home-based outpatient clinical trial on COVID-19 treatments and to identify the early barriers, facilitators and adaptations to the implementation of the trial. We thereby aimed to contribute to improve awareness and preparedness of health systems, healthcare professionals, and scientists for future research on emerging or threatening infectious diseases [20].

## Methods

### Study setting: the Coverage trial

Coverage was a multi-site phase IIb-III outpatient randomized controlled clinical trial which aimed to evaluate the efficacy and safety of drug therapies to reduce the risk of worsening in at-risk individuals aged 50 years and above with early symptomatic COVID-19 infection (less than 7 days) (NCT04356495) [21]. The Coverage trial protocol was submitted to a French Ethics Committee (CPPIDF1-2020-ND45) and to the French National Agency for the Medicines Safety (ANSM) on 31 March 2020. The trial activities started on 16 April 2020 and the first patient enrolment was on 29 July (see Supplementary material 2). First implemented in Bordeaux, the trial had 14 trial centres in 9 French regions in June 2021, each centre having its own operational organization. The trial ended on 3 December 2021.

For this paper, we have focused on the implementation phase of the Coverage trial of the Bordeaux area, where the operative model aimed at reaching people as soon as possible after the onset of their symptoms: dedicated mobile medical teams consisting of a physician (mostly GPs) and a nurse who enrolled patients and conducted follow-up through both face-to-face home visits and telephone assessments. The participants’ GPs were informed about their patient’s trial participation in order to prevent disrupting the routine healthcare pathway of these at-risk (elderly and co-morbid) patients.

In Bordeaux, a large number of personnel volunteered to set-up and implement the Coverage trial, either in addition or instead of their existing professional duties (Table 1). Most medical volunteers had no professional activity at the time due to the lockdown, and little or no research experience. A gymnasium located near the Bordeaux University Hospital (CHU Pellegrin) was used as an "operational base" for the “operational and clinical” team and to store vehicles, as well as medical and disinfection equipment. The trial investigation and coordination unit, based at Bordeaux University and Bordeaux University Hospital participated in the design and implementation of the study from a distance, often in addition to their current professional activities and commitments.

**Table 1:** Stakeholders involved in the Coverage trial implementation between mid-March and end-June 2020, (*n* = 213)

	**Function**	**Role within the trial**	**N**
Trial investigation and coordination team	Sponsors	Promotion of the trial Responsible for the proper conduct of the trial in compliance with existing regulations Financial and administrative oversight of the trial	13
	Methodological centre & Trial Coordination Unit	Contribution to trial protocol, information sheet and informed consent form for patients, design of the electronic case report form, coordination of trial implementation	31
	Researchers	Design of the trial. Supervision and coordination of trial implementation	25
Operational and clinical team (working from the operational office/base)	Study coordinators	Coordination of trial implementation in the field	6
	General practitioners (GP)	Information of eligible patients, consent collection, clinical activity as part of mobile medical teams	14
	Non-medical students and professionals	Administrative support and human resources management	7
	Health students	Logistical support to mobile medical teams	117

### Study design, study population and data collection

We conducted a cross-sectional mixed methods sub-study among stakeholders involved between March and June 2020 in the Coverage trial design, coordination and implementation process. An exhaustive list of stakeholders was drawn by the trial manager and principal investigator (PI). In July–August 2020, all stakeholders were invited by email to respond to an online self-administered 15-min questionnaire (See Supplementary material 3) covering the following domains 1) Sociodemographic characteristics, 2) Experience in clinical research before the Coverage trial, 3) Motivations to take part in the Coverage trial, 4) Experience within the Coverage trial, 5) Perceptions and lessons learned from the Coverage trial experience and more globally regarding outpatient therapeutic trials in emergency epidemic contexts. The majority of the possible answers on experiences and perceptions were based on Likert scales, with the following modalities: “Totally agree”, “Partially agree”, “Partially disagree”, “Totally disagree” and “No opinion”. An email reminder was sent to non-respondents after 10 days and then after 20 days.

A convenience sample of 13 trial stakeholders having been invited to the questionnaire, as well as 1 GP from an emergency service that partnered with the trial team, were interviewed (12 individually and 2 together). Semi-structured interviews were conducted by an interviewer independent from both the investigation and operational teams. They were conducted by phone, in order to respect COVID-19 social distancing measures. The interview guide explored similar themes to the questionnaire (See Supplementary material 4). Interviews lasted for 20 to 40 min. Written notes were taken by the interviewer during the interview.

### Analysis process

For the quantitative data, we computed percentages of “people who agreed” with each statement (merging the “Totally agree” and “Partially agree” modalities) and “people who disagreed” (merging the “Partially disagree” and “Totally disagree” modalities). We analysed responses globally and also stratifying according to the respondent’s profile (members of the “trial investigation and coordination” team versus of the “operational and clinical” team). Missing data were excluded from percentage calculations. All analyses were performed with R v.3.6.1 software. For the qualitative data, written notes were analysed manually and thematically using both a deductive and inductive approach, triangulating between i) initial scientific research questions, ii) predefined themes included in the semi-structured interview guide and iii) recurrent emerging themes discussed by the interviewees. Verbatim from the written interview notes were translated from French to English by the co-authors, and are presented in italic. Quantitative and qualitative data were first analysed separately, then merged and organised by themes.

## Results

### Participants’ characteristics

#### Participants who completed the questionnaire

The questionnaire response rate was 73.6% (156/213 people invited): 72% among trial investigators and coordinators, 100% among GPs, 66% among health students and 82% among other stakeholders. Respondents were 111 (71.1%) from the operational team and 45 (28.9%) from the investigation team (Table 2). Participants were mostly women (66.1%) and 44.2% were under 30 years of age (Table 2). Half of the respondents were students/medical residents (9.0% were from the general practice setting) and 40.0% were working at the university and/or hospital. More than half of participants had no previous experience of participating in clinical research, including 68.0% among those from the operational team (Table 2).

**Table 2:** Profile of Coverage trial stakeholders who responded to the questionnaire, July–August 2020, Bordeaux, France

	Number (%)
**Gender (*n* = *156)***	
Male	52 (33,3)
Female	103 (66,1)
Others	1 (0,6)
**Age *(n* = *156)***	
18–29	69 (44,2)
30–39	43 (27,6)
40–49	28 (17,9)
50 and above	16 (10,3)
**Professional activity (*n* = *156)***	
Hospital students	24 (15,4)
*Medicine*	*15 (9,6)*
*Pharmacy*	*9 (5,8)*
Residents	30 (19,2)
*General practice*	*10 (6,4)*
*Other medical or surgical disciplines*	*12 (7,7)*
*Pharmacy*	*8 (5,1)*
Anaesthesiology nurse students	25 (16,0)
Senior hospital physician/pharmacist	18 (11,5)
General practitioners (GP)	14 (9,1)
Others^a^	45 (28,8)
**Team *(n* = *156)***	
Investigation and coordination	45 (28.9)
Operational and clinical	111 (71.1)
**Previous experience in clinical research *(n* = *147)***	
At least one	69 (46,9)
In outpatient setting(s)	29 (19,6)
In epidemic context(s)	19 (12,9)

^a^CRID (Clinical Research and Innovation Directorate), researchers, engineers, Clinical Research Associates, non-medical or pharmaceutical students, logistician, admin/HR

#### Participants who were  interviewed

Among the 14 participants interviewed, 10 were women and 5 were under 30 years old (Table 3). Four were students/residents, 7 were working within research teams (as clinical research associates, human resources managers, logisticians or methodologists), 2 were locum GPs and 1 was an emergency private medical service GP.

**Table 3:** Profile of Coverage trial stakeholders who were interviewed, July–August 2020, Bordeaux, France

	Number (%)
**Gender (*n* = *14)***	
Male	4 (29)
Female	10 (71)
**Age *(n* = *14)***	
18–29	5 (36)
30–39	5 (36)
40–49	3 (21)
50 and above	1 (7)
**Professional activity *(n* = *14)***	
Hospital students	0
Residents	3 (21)
Anaesthesiology nurse students	1 (7)
Senior hospital physician/pharmacist	3 (21)
General practitioners (GP)	2 (14.5)
Others	5 (36)

### Professional motivations for being involved in the Coverage trial

The initial motivation for participating in the set-up and implementation of the Coverage trial was to feel useful in a time of health crisis (91.9%). This was also reported in the interviews, with the fear of frustration and *guilt of not doing anything to fight the epidemic*, and not being *in the field* as a health professional. Contributing to avoid hospitalization for patients with COVID-19 was also reported as an important motivation during interviews.

83.5% of actors agreed that they were involved in the Coverage trial because of their specific involvement in research or care prior to the outbreak. But half of the participants reported lack of clinical research experience (68% within the operational team; 15% among the investigation team), some of them having to *learn while doing the job* and *adapt to the situation and deal with uncertainties* related to research and regulatory constraints.

### Implementing the Coverage trial in an emergency context

**Fig. 1:**
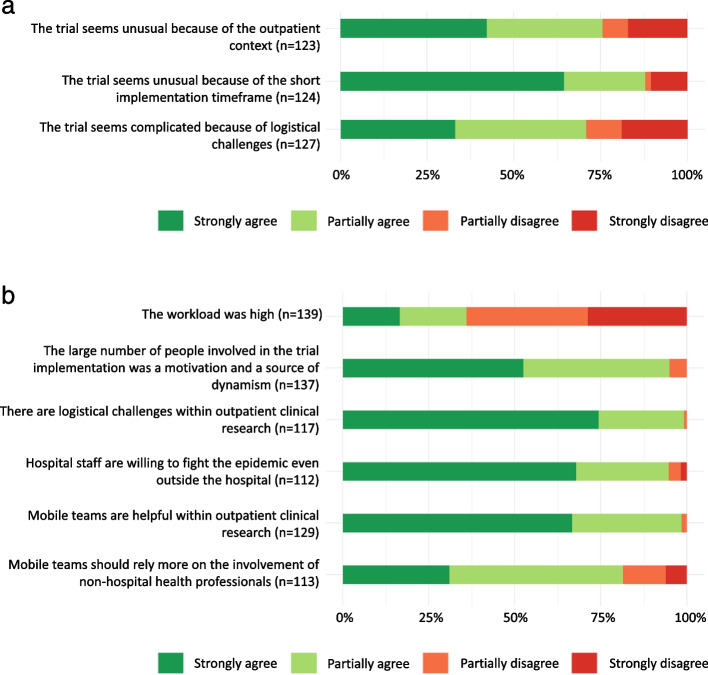
Overall perception of the Coverage trial implementation. (n: number of respondents to the question, i.e. excludes missing responses). **a** Early perceptions of the trial. **b** Perceptions of the operational implementation of the trial

87.9% of respondents, agreed that the Coverage trial was unusual due to its short timeframe for set-up and implementation (Fig. 1a). The interviewees explained that the design, preparation and organization phases of the trial had been condensed, even sometimes superimposed; It indeed took two weeks to secure seed funding, and less than one month went by between the start of writing the protocol and the first day of activity of the trial. The approval of amendments took however longer, and respondents reported a *feeling of lack of flexibility and reactivity* of the regulatory authorities in this emergency context, as well as a *gap between the regulatory requirements and the reality in the field*. During the preparatory phase of the trial, workload was perceived as high by 80% of the investigating team and by 18% among the operational team members.

Most respondents (94.9%) perceived that the large number of actors mobilised in the emergency set-up and implementation of the Coverage trial was something positive (Fig. 1b). This had been a *driving force in the trial*, it encouraged *emulation*, *teamwork and mutual aid*. *Communication between the trial investigation and operational teams* was appreciated. Yet differences in work habits and language, lack of awareness and use of specific tools or procedures, and sometimes communication challenges between the different trial teams, were also reported, often related to the differences in prior training and experience in clinical research.

### Implementing the Coverage trial in an outpatient context

**Fig. 2:**
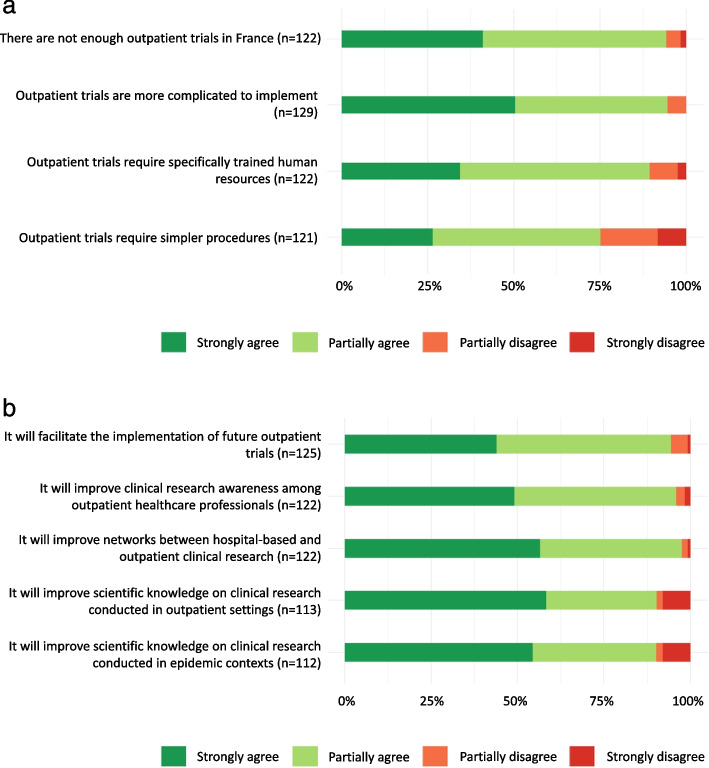
Perceptions of outpatient trials and of the Coverage trial contribution. **a** Perceptions of outpatient trials compared to hospital-based trials. **b** Perceptions on the contribution of the Coverage trial

For 98.4% of respondents, mobile medical teams are useful in out-of-hospital clinical research (Fig. 1b). They suggested that outpatient trials could *help to reach more diverse populations than inpatient trials.* Outpatient trials would also contribute to *maintain patients at home,* thus limiting the risk of disease transmission within health institutions and preventing hospitals from being overwhelmed – thus avoiding depriving more seriously ill people of hospital care. Participants recommended *strengthening research and collaborative practice between GPs and hospital doctors*. Indeed, 81.4% agreed that non-hospital health professionals should be more involved in the early stages of trial design (Fig. 1b). However, participants agreed that trials in outpatient settings require specific professional and research skills (89.3%) (Fig. 2a) and raised specific logistical challenges during implementation (99.1%) (Fig. 1b). Moreover, 94.6% of respondents described the implementation of an outpatient trial as more complicated than a hospital-based trial (Fig. 2a), especially because of the lack of pre-established procedures. The Coverage trial was perceived as useful in encouraging the development of networks between hospital and out-of-hospital clinical research for 97.6% of respondents (Fig. 2b). However, the interviews highlighted a need for *more fluidity* in communication between trial stakeholders, community medicine and primary care medicine partners, particularly better feedbacks on the research and clinical activities carried out within the trial.

### Lessons learned from the Coverage trial for future outpatient clinical trials

According to 94.4% of the respondents, the Coverage trial experience will facilitate the implementation of future outpatient clinical trials (Fig. 2b). During the interviews, the Coverage trial was described as contributing to *de-dramatise* clinical research. But the Coverage trial also highlighted the need to further train GPs in applied research in community medicine and to ensure they can be available for implementing clinical research in outpatient settings. For 95.9% of the respondents, the Coverage trial has raised awareness among out-of-hospital health professionals about participating in clinical studies (Fig. 2b). The Coverage trial was described as a trial that had made it possible to *change practices and advance clinical research in community and primary care medicine* in a situation where *France is lagging far behind other developed countries in this area*. Several stakeholders reported re-thinking about their professional practice and being willing to consider alternative types of clinical trials.

## Discussion

Stakeholders involved in the early implementation phases of the Coverage trial in an epidemic emergency context reported barriers and facilitators related to the construction and organisation of this research, their professional and interpersonal relationships, as well as for the future of outpatient clinical trials.

### Regulating clinical research in times of infectious epidemics and emergencies

The emergency of this rapidly growing pandemic triggered a massive research effort, with more than 2800 trials worldwide, registered as of February 2021 [22]. Sponsors, as well as regulators, ethical bodies and funding agencies also needed to adapt and facilitate this emergency research, with production of new guidance [23], “fast track” evaluation of protocols, or rapid provision of funds. The Coverage trial stakeholders interviewed explained the very short delays in securing seed funding, writing the protocol and starting trial activities, in a context where the average time required to implement a therapeutic trial is usually estimated at between 12 and 18 months [24]. Questions have been raised on the quality of the research approved and implemented under such conditions and several reflections and recommendations shared for the future [22, 25]. In France, the CAPNET “national research priority” label was created early 2020 to accelerate and facilitate COVID-19 clinical research [26], and the Coverage France received that label on 08 December 2020. The need for the right balance between scientific goals and performance, ethicality and adaptation to cultural contexts had already been emphasized during and after the Ebola epidemics [27–29].

Despite having benefited from fast-track processes, many of the stakeholders of the Coverage trial reported that they had faced challenges with constraining regulations and slow administrative processes, which they felt were frustrating in the context of emergency implementation and operational constraints. Among the lessons learned from the Ebola epidemics was the need to “integrate clinical research efforts to epidemic responses and coordinate all actors” to ensure “effective, coordinated and relevant” research timelines prior reaching the epidemic peak [30]. Lessons learned from the COVID-19 pandemic include arguments for “regulatory flexibility”, needed to both protect participants and promote the development of high-quality evidence [31]. Scientists investigating ethics and laws biomedical science have promoted “regulatory agility” for COVID-19 research, with additional resources, specific authorisations put in place and global collaboration [32]. Other recommend rethinking and accepting exemptions to data privacy regulations in times of epidemic outbreaks [33]. As summarised by Janiaud et al., “*pragmatism, integration in clinical care, efficient administration, promotion of collaborative structures, and enhanced integration of existing data and facilities might be several of the legacies of COVID-19 on future randomized trials*” [22]. Our findings can reinforce the need for pre-designed adaptative trial protocols, with specific pre-authorizations, that could be activated in a very short time as soon as sufficient data is available to support the evaluation of one or several treatments, as outlined in Sigfrid et al. [9].

### Implementing future out-of-hospital therapeutic trials

The set-up and early implementation of the Coverage trial in the COVID-19 outbreak context involved and/or requisitioned the involvement of a large number of actors from several disciplines, which was perceived as a source of dynamism by interviewees; this has been reported in another study in the USA [34]. However, while the motivations of young clinicians and GPs to be involved in clinical research, to train on-the-job, and to participate in the Coverage trial implementation was praised by some of the stakeholders interviewed, others questioned whether this lack of prior training and experience has been an obstacle to the implementation of the trial. Within the Coverage trial, monitoring good clinical research practices (compliance with regulations and participant safety) relied on the expertise of hospital-based clinical research professionals (Bordeaux University Hospital Directorate of Clinical Research and Innovation; Clinical Trial Unit; hospital-based clinicians already familiar with and trained to research). But as recommended by several stakeholders interviewed, the experience from the Coverage trial highlights the need for improving access to clinical research training; it would indeed help to improve the rapid mobilisation of professionals, whether hospital-based or not, in order to respond to future epidemic crises and associated emergency research. Within the 2020 report on the “missions of clinical trials in an epidemic context” [35], French experts suggested a national action plan for research (“plan blanc”), as well as the constitution of a mobilisable health research “reserve”.

Beyond human resources management, several operational strategies may facilitate outpatient trials, as recently reported by an American study, with the generalization of electronic consent, teleconsultation, or the delivery of experimental drugs by couriers [35]. Other strategies include (i) fast-track training on research methodology and procedures, (ii) development of research methodology adapted to general practice constraints (for example lack of time, human resources shortages), and (iii) organisation of research networks between hospital and community medicine [36]. Promoting such innovations, and contributing to overall resilience in research practices, will improve high quality research practices and findings [37]. It is of particular importance in a context where the World Health Organization (WHO) itself is calling for improved research preparedness for the next pandemic after COVID-19 [20, 38].

### Strengths and limitations

This study is one of the firsts to describe the set-up and early implementation of an outpatient clinical trial in an emergency epidemic context. The mixed methods design provided both quantitative measurements of main perceptions among trial stakeholders and also qualitative insights into individual experiences, perspectives and feelings. This study was conducted by a group of researchers with different responsibilities within the Coverage trial: co-primary investigators (DM, XA), co-investigators (AD, RO, JPJ), investigators and research assistants on the acceptability and feasibility component (JOG, ML, CG, MP), one of whom was also in charge of field operations (CG). Though it could be outlined as a risk of bias in data collection and analysis, it also contributed to a comprehensive assessment of stakeholders’ experiences, and discussion of the current study results from multiple perspectives.

Yet, our study faces some limitations. First, as the study was implemented at the very beginning of the Coverage trial – thus before the first patient inclusions (consistent with the epidemic curve in France at the time) – the experiences shared within this paper do not capture the barriers and facilitators of the trial implementation during recruitment period. However, the feasibility and acceptability of the trial and of the outpatient and home-based model is currently being investigated within the Coverage ACCEPT sub-study conducted among trial participants and healthcare providers. Second, only stakeholders involved in the trial coordination and implementation were interviewed here; the perceptions and experiences of external stakeholders, such as other GPs, clinicians, or members of regulatory agencies may have provided valuable insight. Third, even if the response rate for the quantitative survey is relatively high (even if not optimal among health students), the rate of missing answers was > 25% for some questions, and the reasons of missing value were not well documented (it could be because people did not know how to answer the question, or did not want to answer it, or were not concerned by the question). Forth, interviews were not recorded; and written notes were taken while conducting the interviews. This may have limited the interviewers’ attention and capacity to explore in detail certain issues. Of note only verbatim interview extracts are presented in the paper. Finally, as it is one of the first studies on the subject, there are very few peer-reviewed papers published internationally to which we can compare and contrast our data to, and we thus used national French documents to interpret our results, i.e., maybe only relevant in the French or European contexts. The question of the implementation of outpatient trial in emergency contexts should be further explored in different settings worldwide.

## Conclusions

In an emergency epidemic context, the implementation of an outpatient clinical trial with at-home follow-up was perceived as relevant and innovative although requiring important adaptations to usual professional responsibilities and standard research procedures. While many ongoing COVID-19 research studies focus on finding treatments or assessing the many severe consequences of the pandemic and the measures implemented to prevent ongoing transmission, we also need research focusing on improving awareness and preparedness of health systems, of healthcare professionals, and of scientists, to face such crises and learn for the short- and longer-term. Lessons learned from the Coverage trial underline the need for improved networks between hospital and community medicine, and call for a dedicated and reactive outpatient research platform on emerging or threatening infectious diseases. These study findings may contribute to the structuration/restructuration of infectious disease research conducted in emergency epidemic contexts.

## Supplementary Information

**Additional file 1: Supplementary material 1.** Coverage study group. **Supplementary material 2.** Implementation phases of the Coverage trial in Bordeaux area, according to the Covid-19 epidemic evolution in France (March-November 2020). **Supplementary material 3.** Coverage's stakeholder self-administered questionnaire. **Supplementary material 4.** Coverage stakeholders interview guide.

## Data Availability

The data that support the findings of this study are available from University of Bordeaux/CHU Bordeaux but restrictions apply to the availability of these data, which were used under license for the current study, and so are not publicly available. Data are however available from the authors upon reasonable request and with permission of Pr X Anglaret and Dr Joanna Orne-Gliemann.
