# Supplementary material for: Implementing an outpatient clinical trial on COVID-19 treatment in an emergency epidemic context: a mixed methods study among operational and research stakeholders within the Coverage trial, Bordeaux (France)
Source: Arch Public Health. 2022 Dec 3;80:245. doi: 10.1186/s13690-022-00999-9 (PMC9719237; doi:10.1186/s13690-022-00999-9)
Supplement: Supplementary file 1 — Additional file 1: Supplementary material 1. Coverage study group. Supplementary material 2. Implementation phases of the Coverage trial in Bordeaux area, according to the Covid-19 epidemic evolution in France (March-November 2020). Supplementary material 3. Coverage's stakeholder self-administered questionnaire. Supplementary material 4. Coverage stakeholders interview guide. [file 13690_2022_999_MOESM1_ESM.pdf]

Supplementary material

- *Supplementary material 1. Coverage study group*
- *Supplementary material 2. Implementation phases of the Coverage trial in Bordeaux area, according to the Covid-19 epidemic evolution in France (March-November 2020).*
- *Supplementary material 3. Coverage stakeholders self-administered questionnaire.*
- *Supplementary material 4. Coverage stakeholders interview guide*

Supplementary material 1. Coverage study group

COVERAGE Study Group

Version 4.0 – 16 November 2021

Asterisk (*) indicates staff who left during the trial.

- ***Trial Steering Committee (TSC):***

Voting members*:* X. de Lamballerie (Chair), X. Anglaret, L. Atlani-Duault, C. Begue, F. Chauvin*, J. Chastang, D. Darmon, T. Darnaud, P-L. Druais, V. Dubee, J. Dupouy, A. Duvignaud, A. Gimbert, V. Journot, R. Landman, D. Lebeaux, B. Lefèvre, E. Lhomme, A. Makinson, D. Malvy, F. Mentré, J-F. Michel*, M. Molimard, J-L. Montastruc, JM Naccache, J. Orne Gliemann, L. Piroth, C. Rat, L. Richert, C. Roussillon, O. Saint Lary, R. Thiebaut, L. Weiss, L. Wittkop

Non-voting members*:* S. Bouchet, C. Cazenave*, S. Conde*, A. Cremer, S. Djabarouti, C. Gil Jardine, L. Hardel, J-P. Joseph, M-E. Lafon, J. Le Bel, L. Letinier*, S. Marchi, L. Moinot*, A. Montoya-Ferrer, JD. Nguyen, R. Onaisi, I. Pellegrin, T. Pistone, D. Poitrenaud, T. Schaeverbeke*

- ***Data Safety Monitoring Board (DSMB):*** D. Costagliola (Chair), E. Bellissant*, G. Gavazzi, C. Locher, A-M. Taburet, P. Tattevin, S. Walker

- ***Coordinating Unit***

CIC-EC 1401/EUCLID: L. Richert (head), F. Allais*, S. Canete, N. Chaghil, S. Daoui*, G. Dupouy*, L. Esterle*, V. Favreau, A. Gelley, S. Gillet*, L. Hardel, M. Kanté*, E. Lhomme, S. Martiren*, L. Moinot*, L. Pinoges, E. Rouch*, C. Schwimmer, R. Sitta, M. Termote*, C. Wallet, L. Wittkop

MEREVA: X. Anglaret (head), E. Balestre, A. Beuscart*, C. Bonnier*, C. Cazes*, C. Chazallon, G. Clouet, M. Daures*, D. Gabillard, G. Habiyambere*, V. Journot, M. Loniewski, S. Karcher, J. Le Carrou*, O. Marcy, V. Murat, J. Orne Gliemann, M. Plazy

- ***CHU Bordeaux, trial sponsor:*** G. Duluc (head), D. Arma, P. Beaufrère, J. Belcastro*, T. Brice, P. Cassai, S. Desjardins, J. Durrieu, S. Georgevail, A. Gimbert, S. Marchi, V. Marty, F. Nacka, R. Pinilla, P. Poulizac, S. Regueme*, M. Rousset, C. Roussillon, F. Salvo*, J. Soria, S. Vautrat

- ***Coordinating Pharmacy:*** S. Djabarouti (head), B. Ghezzoul, L. Delignac*, P. Etienne, V. Fulda, M. Gigan*, C. Langlade, J. Le Chanjour*, P. Marque, P. Mora, C. Plessis*, B. Sourisseau

- ***Laboratory responsible of final analysis :***

Bordeaux: I. Pellegrin (head), S. Bouchet, C. Cognet, I. Garrigue, J. Jeanpetit, M-E. Lafon, A. Pouzet, A. Tarricone, P. Trimoulet, A. Voldoire

Créteil (sub-study): C. Lacabaratz (head), H. Hocini

- ***Bordeaux study site:***

Investigating physicians: A. Duvignaud (principal investigator), C. Bez, E. Bironneau*, E. Collomb, A. Contamin, C. Dubourdieu,, N. Faure*, M. Galinski, P. Gibaud*, C. Gil-Jardine, J. Guillot-Warin*, C. Lebouc, A. Leger, V. Lengline, C. Loizeau, M. Mayenc, N. Merle, H. Nadiri-Kahraman, D. Nguyen, M. Odorico, R. Onaisi, T. Pistone, F. Sacher*, J. Scandella, F. Velardo, AS. Wiet

Coordination: X. Anglaret (head), T. Boudon, O. Bouissière, R. Brégéras*, N. Broennec*, S. Condé*, C. Gazille*, C. Grenier*, G Kouame*, C. Martins-Calado, J-B. N'Takpe*, Z. Pascual*

- ***Dijon Study site***:

Investigating physicians: L. Piroth (principal investigator), S. Court Devilliers, J. Darley, F. Ducherpozat, N. Eberard, JL. Faure, H. Portier, R. Rochelet, C. Ruffino, R. Thevenoud

Coordination: C. Binquet, TT. Creusvaux-Nguyen, N. Desbiolles, A. Grattard, A. Lamotte Felin, L. Rossye, C. Schaeffer, F. Silvestre, C. Simonel,

- ***Nancy Study site:***

Investigating physicians: B. Lefèvre (principal investigator), G. Baronnet, E. Baux, C. Di Santolo, M. Ferry, F. Goehringer

Coordination: C. Daguin, E. Dauchy, M. Gilg, P. Rossignol

- ***Toulouse Study sites***:

Investigating physicians: J. Dupouy (principal investigator, Pins Justaret), A. Boucault (principal investigator, La Providence), L. Gimenez (principal investigator, Colomiers), C. Burguier, M. Couderc, C. Fradet, A. Gervais, J. Lavergne, C. Landon, S. Mathe, B. Ortala

Coordination and monitoring: N. Ainaoui, S. Auriac, S. Flasquin, C. Fraysse, M. Gabriel, P. Gauteul, J. Germain, F. Gross, C. Lebely*, C. Riviere*, D. Robert, C. Thalamas

- ***Bastia Study site***:

Investigating physicians: T. Darnaud (principal investigator), D. Poitrenaud*

Coordination: I. Giusti, S. Provent

- ***Montpellier Study site:***

Investigating physicians: A. Makinson (principal investigator), A. Montoya-Ferrer

Coordination and monitoring: J. Battery, N. Coux, L. Crantelle, F. Galtier, C. Martin*, MC Picot

- ***Paris Study sites:***

Monitoring (Institut de Médecine et d'Epidémiologie Appliquée - IMEA): R. Landman (head), K. Amat, A. Benalicherif, B. Sylla

  - Groupe Hospitalier Paris Saint Joseph (GHPSJ) :

***Investigating physicians*:** JM. Naccache (principal investigator GHPSJ), J. Jouveshomme, E. Devaud (principal investigator Cergy-Pontoise)

***Coordination*:** N. Ben Nasr, R. Monkam, E. Sacco, S. Rulle

  - Collège National des Généralistes Enseignants (CNGE):

***Investigating physicians*:** J. Le Bel (principal investigator), J. Chastang, A. Nguyen, O. Saint-Lary

***Coordination*:** I. Giraud

- ***Nantes/Angers Study sites:***

***Investigating physicians*:** C. Begue (principal investigator Angers), C. Rat (principal investigator Nantes), C. Bouve, C. Huard, S. Morgand

***Coordination*:** E. Guegan, D. Fairier, A. Loiez

- ***Others contributors:*** T. Alcouffe, V. Briand, S. Coudray, D. Charles*, H. Jacquet, C. Levy-Marchal, J. Raude, P. Vellozzo, P. Whithenay*

Supplementary material 2. Implementation phases of the Coverage trial in Bordeaux area, according to the Covid-19 epidemic evolution in France (March-November 2020).

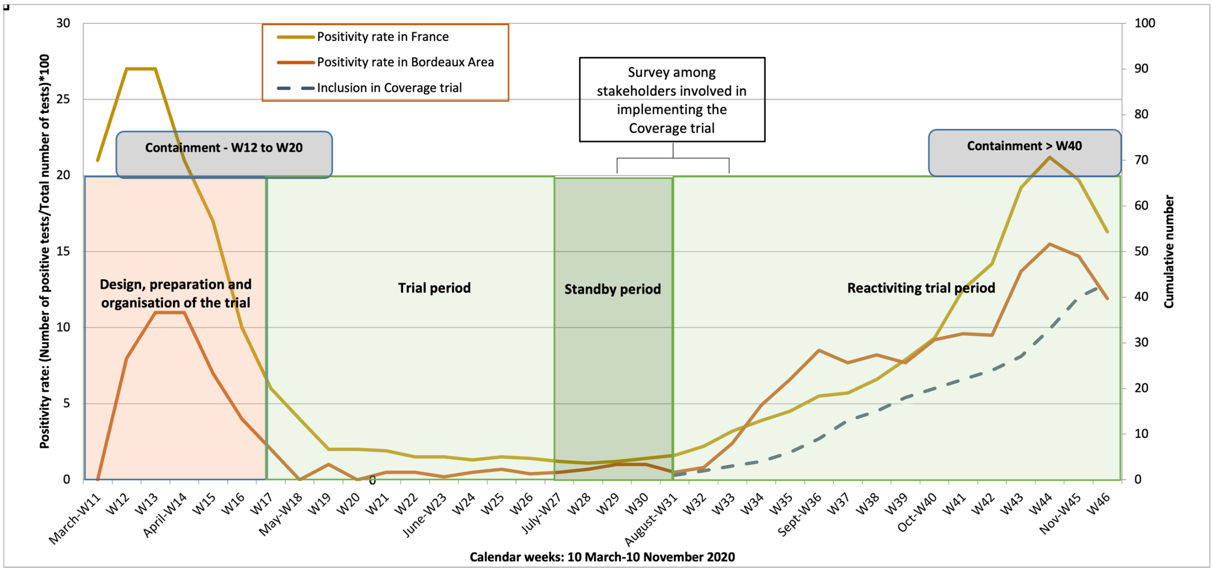

Supplementary material 3. Coverage stakeholders self-administered questionnaire.

This questionnaire is intended for people who contributed to the Coverage trial between March 15 and June 30, 2020, in Bordeaux.

Coverage was set up in a *very short time*, in a context of *epidemic threat* and in an *out-of-hospital* setting. These three characteristics implied several original challenges, on which we have little previous experience. We believe that the perception of the stakeholders who participated in this project could provide useful lessons for the future.

The questionnaire is anonymous. The collection tool does not record IP addresses. Your participation is free. It will take about 15 minutes to complete. There are no right or wrong answers. No value judgement on the Coverage trial or its actors will be made on the basis of these answers. Our approach is just to describe the different points of view, to think about the future. When the answers are presented by "type of actor", it will be in very large groups, so that no individual opinion can be identified. If any questions seem unclear, inappropriate or if you have no opinion on them, you can use the "no opinion" boxes. All questions are single choice.

The responses will be analyzed as part of a master's thesis in public health. The results will be communicated to the entire Coverage team at the end of September. No use will be made of them without discussion and agreement within the framework of the trial's scientific council.

.

The deadline for completing the survey is July 26, 2020.

Thank you very much for your participation!

I understand and I participate (=> access to the questionnaire) I do not wish to participate

BEGIN QUESTIONNAIRE

I Your profile

1 **Age**

- ☐ 18-29 years old
- ☐ 30-39 years old
- ☐ 40-49 years old
- ☐ 50 and over

2 **Gender**

- ☐ Man
- ☐ Woman
- ☐ Other

3 **(Professional) Activity**

- ☐ Hospital student
  - **Field**:
    - ☐ Pharmacy (all fields)
    - ☐ Medicine
  - **Intended Specialty:**
    - ☐ General medicine
    - ☐ Other medical or surgical specialty
    - ☐ I do not know
- ☐ Intern
  - **Field or specialty**:
    - ☐ General medicine
    - ☐ Other medical or surgical specialty
    - ☐ Pharmacy (all fields)
- ☐ Outpatient physician
- ☐ Hospital physician or pharmacist (excluding DRCI)
- ☐ Nurse
- ☐ Other (doctor or pharmacist DRCI, researcher, engineer, ARC/TEC, admin/HR; trainee license or master; logistician)

II Your experience in clinical research prior to participating in Coverage

The following questions are about your experience of active participation in clinical research during your studies and/or your professional activity.

- ***Active participation:*** *participation with specific tasks and a well identified place in the organization chart.*
- ***Clinical study***: medical research on human beings.
- ***Therapeutic trial***: clinical study evaluating the dose, efficacy and/or safety of a health product (drug or device) in healthy or sick volunteers.
- ***Hospital-based clinical trial or study***: a study in which recruitment takes place in a hospital, either as an outpatient or an inpatient.
- ***Outpatient clinical trial or study***: a study in which recruitment takes place in the community (medical practice, health center, residence for the elderly, EHPAD).

4 **Did you have at least one experience of <u>active</u> participation in clinical studies and/or therapeutic trials before Coverage?**

- ☐ Yes
- ☐ No

If not, go to question 5.
If yes, answer questions 4.1, 4.2 and 4.3:

4.1 In what context?

4.1.1 In a hospital setting ☐Yes☐ No

If yes, how many hospital-based studies have you participated in?

4.1.1.1 *Therapeutic trials:*

- ☐ 1
- ☐ 2
- ☐ 3 or more

4.1.1.2 *Other types of clinical studies:*

- ☐ 1
- ☐ 2
- ☐ 3 or more

4.1.2 In a non-hospital setting ☐Yes☐ No

If yes, how many out-of-hospital studies have you participated in?

4.1.2.1 *Therapeutic trials:*

- ☐ 1
- ☐ 2
- ☐ 3 or more

4.1.2.2 *Other types of clinical studies:*

- ☐ 1
- ☐ 2
- ☐ 3 or more

4.2 Did at least one of these studies take place in France or another high-income country?

- ☐ Yes, at least one
- ☐ No, none

4.3 Had at least one of these studies taken place in the context of a threatening acute epidemic *(e.g. SARS, H5N1, Chikungunya, Ebola, Lassa, Zika, etc.)*?

- ☐ Yes, at least one
- ☐ No, no

III Your experience of Covid-19 before participating in Coverage

The following questions are for the period between the start of containment (March 17, 2020) and the start of your participation in the Coverage trial.

5 **At the beginning of the lockdown, how did your workstation change?**

- ☐ I continued to work at my usual place of work
- ☐ I continued to work but from home
- ☐ I stopped working (interrupted job/internship, or new job/internship delayed)
- ☐ Other situation --> Specify _ _ _ _ _ _ _ _ _ _ _ _ _ _ _ _ _

6 **At the beginning of the lockdown, were you concerned about:**

	Quite concerned	Somewhat concerned	Not really worried	Not at all worried	No opinion or not applicable
6.1 **The consequences of the virus on your own health**	☐	☐	☐	☐	☐
6.2 **The consequences of the virus on the health of others**	☐	☐	☐	☐	☐
6.3 **The social and economic consequences of the epidemic**	☐	☐	☐	☐	☐
6.4 **Disruptions to your working conditions or internship**	☐	☐	☐	☐	☐
6.5 **Another concern**	☐	☐	☐	☐	☐
6.5.1 If other, specify: _					

7 **At the beginning of the containment, did you think that the return to the previous situation *(i.e. organization of work and life in society similar to those before the beginning of the epidemic)* would be done in:**

- ☐ Less than a month
- ☐ 1-3 months
- ☐ 4-6 months
- ☐ More than six months
- ☐ Never
- ☐ Don't know

IV Your participation in the Coverage trial

The following questions are about the conditions and experiences of your participation in Coverage.

8 **When did you start <u>actively participating</u> in the Coverage trial *(actively participating = you attended a training session with the intention of participating in Coverage, or you were given one or more tasks to perform for Coverage, either remotely or face-to-face)***

- ☐ Before April 13 (before the opening of the Coverage base at Chaban-Delmas)
- ☐ Between April 14 (from the opening of the Coverage base at Chaban-Delmas) and May 31
- ☐ From June 1st

9 **<u>Initially,</u> what were your motivations for participating in Coverage:**

	Totally agree	Somewhat agree	Somewhat disagree	Not at all in agreement	No opinion or not applicable
9.1 **My job/position meant that I was asked to work on the project**	☐	☐	☐	☐	☐
9.2 **To validate an internship**	☐	☐	☐	☐	☐
9.3 **To add experience to my professional/student background**	☐	☐	☐	☐	☐
9.4 **To feel useful in times of health crisis**	☐	☐	☐	☐	☐
9.5 **To reach people already involved in the project**	☐	☐	☐	☐	☐
9.6 **For another reason**	☐				
9.7 **If other, specify: _**					

10 **What were <u>your first impressions</u> of the Coverage trial (*first impressions = your impressions in the first few days of your active participation in the trial, regardless of how these impressions may have changed later)***

	Totally agree	Somewhat agree	Somewhat disagree	Not at all in agreement	No opinion or not applicable
10.1 **The trial was unusual (compared to other studies you may have participated in if you had previous experience, or to your idea of clinical research if you had no previous experience)**	☐	☐	☐	☐	☐
If "strongly disagree", go to question 10.2<br>*If "strongly agree, somewhat agree, or somewhat disagree," please explain* why:					
10.1.1 Because it is carried out in an out-of-hospital setting	☐	☐	☐	☐	☐
10.1.2 Due to the short implementation time (threatening epidemic)	☐	☐	☐	☐	☐
10.1.3 Because of the risk for the personnel (*transmission of the virus by going to the home of potential cases*)	☐	☐	☐	☐	☐
10.1.4 Because of the risk to participants (*transmission of the virus from one home to another home of potential cases*)	☐	☐	☐	☐	☐
10.1.5 Due to the strong mobilization of the participants	☐	☐	☐	☐	☐
10.1.6 For other reasons ☐					
10.1.6.1 *If other, specify: _*					
10.2 **The trial was complicated (referring to other studies you may have participated in if you had previous experience, or to your idea of clinical research if you had no previous experience)**	☐	☐	☐	☐	☐
If "strongly disagree", go to question 10.3<br>*If "strongly agree, somewhat agree, or somewhat disagree," please explain why*:					
10.2.1 Due to complex logistics (shortage of material, complex circuits, multiple teams...)	☐	☐	☐	☐	☐
10.2.2 Because of the risk of infection (requiring the implementation of protective measures for equipment and people)	☐	☐	☐	☐	☐
10.2.3 Because of the difficulty of recruiting people at home	☐	☐	☐	☐	☐
10.2.4 Due to the implementation of self-monitoring of the participants	☐	☐	☐	☐	☐
10.2.5 For other reasons ☐					
10.2.5.1 *If other, specify: _*					
10.3 **It was going to be a tough try to make it to the end:**	☐	☐	☐	☐	☐
If "strongly disagree", go to question 1110.3<br>*If "strongly agree, somewhat agree, or somewhat disagree," please explain why*:					
10.3.1 Because the epidemic would not last long enough	☐	☐	☐	☐	☐
10.3.2 Because the response time of the authorities would be too long	☐	☐	☐	☐	☐
10.3.3 Because it would be difficult to identify potentially includible people	☐	☐	☐	☐	☐
10.3.4 Because it would be difficult to convince people at home to participate	☐	☐	☐	☐	☐
10.3.5 Because it would be difficult to convince referring physicians to participate	☐	☐	☐	☐	☐
10.3.6 For other reasons ☐					
10.3.6.1 *If other, specify: _*					

11 **In total, for the entire period from March 15 to June 30, *approximately* how much time did your activity within Coverage represent (in cumulative duration expressed in days or weeks)?**

I___I___I days or I___I___I weeks

12 **Over this period, on average, your working time per day spent on Coverage was:**

- ☐ One hour or less
- ☐ Between 1 hour and half a day
- ☐ Between half a day and 8 hours
- ☐ More than 8 hours

13 **Did you take part in Coverage:**

- ☐ Always or mostly from home
- ☐ Always or mostly in person (Chaban base, CHU [including UDH, Ville hop])
- ☐ Remote from and in person

14 **What was your place in the Coverage organization chart?**

- ☐ Member of the promotion team or CMG (DRCI, Euclid, Mereva, ARC base)
- ☐ Mobile teams (doctors, nurses, drivers)
- ☐ Investigating physicians (other than those of the EMMs: infectiologists, pharmacologists, biologists, cardiologists, emergency physicians, etc.)
- ☐ Regulation, Partnership, City-Hop, UDH
- ☐ Logistics, Medical Resources, Admissions, Biology, Medication

15 **As part of Coverage, have you had face-to-face contact with potential participants (as part of a screening or inclusion visit)?**

- ☐ Yes
- ☐ No

16 **As part of Coverage, have you been in telephone contact with potential participants (for information, or to arrange for screening or an inclusion visit)?**

- ☐ Yes
- ☐ No

17 **As part of Coverage, have you been in contact with physicians (referring or coordinating) or other health care professionals outside of the Coverage teams?**

- ☐ Yes
- ☐ No

18 **As a result of your participation, would you say that: [Feedback]**

	Yes, I think so.	Somewhat agree	Somewhat disagree	No, I don't think so at all	No opinion or not applicable
18.1 **The objective of the trial was to**	☐	☐	☐	☐	☐
18.2 **Video conferencing between teams has been :**					
18.2.1 An asset for a quick implementation during the containment period	☐	☐	☐	☐	☐
**18.2.2** An additional source of difficulty	☐	☐	☐	☐	☐
18.3 **The large number of people on the team was :**					
**18.3.1** A richness and a source of dynamism	☐	☐	☐	☐	☐
18.3.2 An additional source of complication	☐	☐	☐	☐	☐
18.4 **The workload in Coverage was heavy (in absolute terms)**	☐	☐	☐	☐	☐
If "strongly disagree", go to question 18.5<br>*If "strongly agree, somewhat agree, or somewhat disagree," please explain* why:					

**18.4.1** The workload was *too* heavy *and* could have been lighter	☐	☐	☐	☐	☐
**18.4.2** The workload was heavy *but* it was unavoidable given the situation	☐	☐	☐	☐	☐
18.5 **Coverage's CRF was complicated (in absolute terms)**	☐	☐	☐	☐	☐
If "strongly disagree", go to question 18.6<br>*If "strongly agree, somewhat agree, or somewhat disagree," please explain why:*					
18.5.1 The CRF was *too* complicated *and* could have been simplified	☐	☐	☐	☐	☐
18.5.2 The CRF was complicated *but* justified	☐	☐	☐	☐	☐
18.6 **The experimental drugs used were not suitable for a home trial**	☐	☐	☐	☐	☐
If "strongly disagree", go to the next question<br>*If "strongly agree, somewhat agree, or somewhat disagree," please explain why:*					
18.6.1 The number of arms was too high	☐	☐	☐	☐	☐
18.6.2 Some drugs had too many non-inclusion criteria	☐	☐	☐	☐	☐
18.6.3 Some medications required too much monitoring	☐	☐	☐	☐	☐
18.7 **The setting up of the base in the Chaban-Delmas stadium was an interesting experience**	☐	☐	☐	☐	☐
If "strongly disagree", go to question 19<br>*If "strongly agree, somewhat agree, or somewhat disagree," please explain* why:					
18.7.1 Because it showed the importance of logistical issues in this type of circumstances	☐	☐	☐	☐	☐
18.7.2 Because it illustrated the willingness of hospital professionals to take action on the epidemic outside the hospital	☐	☐	☐	☐	☐
18.7.3 Because it illustrated the willingness to mobilize institutions and individuals (outside of the hospital and academic communities)	☐	☐	☐	☐	☐

IV What you learned from it

The following questions are about your views on Coverage, as well as on outpatient therapeutic trials in general, after your participation in the trial.

19 **As a result of your participation in Coverage, would you say that: [opinion of out-of-hospital trials in general]**

	Yes, I think so.	Somewhat agree	Somewhat disagree	No, I don't think so at all	No opinion or not applicable
19.1 **There are not enough therapeutic trials in the out-of-hospital setting in France**	☐	☐	☐	☐	☐
19.2 **Compared to hospital-based trials, out-of-hospital trials :**					
19.2.1 Are more complicated to realize for the team in charge of the test	☐	☐	☐	☐	☐
19.2.2 More risky for patients	☐	☐	☐	☐	☐
19.2.3 Are more complicated to explain/accept to patients	☐	☐	☐	☐	☐
**19.2.4** Require regulatory adaptations	☐	☐	☐	☐	☐
**19.2.5** Need simpler procedures	☐	☐	☐	☐	☐
**19.2.6** Need a simpler CRF	☐	☐	☐	☐	☐
**19.2.7** Requires additional human resources	☐	☐	☐	☐	☐
**19.2.8** Need specially trained human resources for this purpose	☐	☐	☐	☐	☐
19.3 **The concept of "mobile teams" in support of out-of-hospital research:**					

19.3.1 is useful in outpatient clinical research	☐	☐	☐	☐	☐
19.3.2 is too complicated and/or too expensive	☐	☐	☐	☐	☐
19.3.3 is interesting but still needs to be improved	☐	☐	☐	☐	☐
19.3.4 should rely more on the involvement of non-hospital health professionals					

20 **Following your participation in Coverage, would you say that? [Perspectives]**

	Yes, I think so.	Somewhat agree	Somewhat disagree	No, I don't think so at all	No opinion or not applicable
20.1 **The Coverage trial will succeed in including at least 90 participants before the end of the epidemic (90=number needed for the 1ère interim analysis)**	☐	☐	☐	☐	☐

21 **As a result of your participation in Coverage, do you feel that: [Secondary benefits]**

	Totally agree	Somewhat agree	Somewhat disagree	Not at all in agreement	No opinion or not applicable
21.1 **If it fails to include the number of participants needed to conclude on the effectiveness of the treatment before the end of the epidemic, the Coverage trial will still have been useful**	☐	☐	☐	☐	☐
If "strongly disagree", go to the next question<br>*If "strongly agree, somewhat agree, or somewhat disagree," please explain* why:					
21.2 **Because, regardless of its primary outcome, a trial such as Coverage can also develop or strengthen scientific knowledge about:**	☐	☐	☐	☐	☐
21.2.1 On therapeutic trials *in general*	☐	☐	☐	☐	☐
21.2.2 On therapeutic trials in the out-of-hospital setting *in particular*	☐	☐	☐	☐	☐
21.2.3 On therapeutic trials *during epidemics*	☐	☐	☐	☐	☐
21.2.4 On the organization of the follow-up in their place of living of people with a suspected or confirmed COVID-19	☐	☐	☐	☐	☐
21.2.5 On another point	☐	☐	☐	☐	☐
21.2.5.1 Specify: _					
21.3 **Because, regardless of its primary outcome, the experience gained from Coverage may facilitate the implementation of future studies:**	☐	☐	☐	☐	☐
If "strongly disagree", go to the next question<br>*If "strongly agree, somewhat agree, or somewhat disagree," please explain* why:					

21.3.1 Promoting awareness among out-of-hospital health professionals about participating in *clinical research studies*	☐	☐	☐	☐	☐
21.3.2 Promoting awareness among *young* health professionals in training to participate in *clinical research studies*	☐	☐	☐	☐	☐
21.3.3 Promoting public awareness of participating in *clinical research studies*	☐	☐	☐	☐	☐
21.3.4 By promoting the development of networks between *hospital* and *non-hospital clinical research*					
21.3.5 Other possible benefits	☐				
21.3.5.1 *Specify: _*					

22 **As a result of your participation in Coverage, would you say that: [Limitations]**

	Totally agree	Somewhat agree	Somewhat disagree	Not at all in agreement	No opinion or not applicable
22.1 **Has Coverage not sufficiently positioned itself from the outset in a national or international research dynamic?**	☐	☐	☐	☐	☐
22.2 **Coverage did not sufficiently anticipate the recruitment capacities related to the dynamics of the epidemic?**	☐	☐	☐	☐	☐
22.3 **Coverage did not sufficiently anticipate the difficulty of recruitment in urban medicine, independently of the dynamics of the epidemic?**	☐	☐	☐	☐	☐
22.4 **Coverage represents a *substantial financial risk (*i.e. risk of costing too much in human and financial resources compared to what it could bring)**	☐	☐	☐	☐	☐
22.1 **Coverage has other weak points**	☐				
22.1.1 Specify: _					

23 **In conclusion, if you had 1-3 words or phrases to summarize your experience in Coverage, what would they be**?

(Avoid making sentences)

**1.**	_ _
**2.**	_ _
**3.**	_ _

Thank you very much for your participation. All the results of this survey will be communicated at the end of September 2020 to all participants.
For any further information on this questionnaire, you can contact me by email: carine.grenier.coverage@chu-bordeaux.fr

Supplementary material 4. Coverage stakeholders interview guide

	Sponsor	Methodologists	Investigating Physicians	Mobile team	Partners	Interns/ residents	Coordination	Liberal nurse	General practitioner
Before we start talking about your involvement in the COVERAGE trial, can you tell me about your experience with clinical trials?<br>• In what contexts? Epidemic emergencies? Out of hospital (ambulatory?)?<br>• With which type partner/association?		**X**	**X**	**X**	**X**	**X**	**X**	**X**	**X**
Can you tell me about your role in the COVERAGE trial?<br>• Theory/reality?<br>• Were your assignments adapted to your job profile? To your expectations? To what you were told?<br>• Did you enjoy this role?	**X**	**X**	**X**	**X**	**X**	**X**	**X**	**X**	**X**
Can you tell me about your feelings about the screening or pre-inclusion process in COVERAGE?<br>• Risk of infection for one self<br>• Risk of spreading the virus oneself or through equipment used, if entering the home of presumptive cases?<br>• Impact on care? patient-caregiver relationship?<br>• Home-based screening or at hospital?<br>--------------------<br>If you have referred patients, can you tell me about their reactions?<br>• Positive reactions, motivation to be tested by Coverage<br>• Negative reactions, reluctance				**X**		**X**		**X**	**X**
Can you tell me what led you to participate in this COVERAGE trial?<br>• Professional, personal motivations?		**X**	**X**	**X**	**X**	**X**	**X**	**X**	**X**
What is your perception of the COVERAGE trial, which was designed and conducted in an emergency setting and in patients' homes? What is your experience?	**X**	**X**	**X**	**X**	**X**	**X**	**X**	**X**	**X**

• Experience + or - with planned or unplanned events during trial implementation<br>• Difficulties & facilities encountered? Why, how?<br>• Comparison with past experience (if applicable)<br>• Multitude of actors→ Experience & feelings (Field / regulatory issues)<br>• Role of each person +/- well defined -> Experience and feelings<br>• Among non-experienced clinicians: experience<br>• Home / Out of Hospital Context<br>• Emergency context (epidemic health crisis situation / rapid installation)									
To date, no inclusion has taken place, how do you feel about this?<br>• What if inclusions had started from the beginning of the trial?	**X**	**X**	**X**	**X**	**X**	**X**	**X**	**X**	**X**
Finally, do you think this experience will have an influence on your future practice?<br>• Why?<br>• In what sense: positive/negative?<br>• In what, how?<br>• What about a possible 2nd wave of Covid? What will happen	**X**	**X**	**X**	**X**	**X**	**X**	**X**	**X**	**X**
